# Infant Mental Development Index: Hu et al. Respond

**Published:** 2007-04

**Authors:** Howard Hu, Martha María Téllez-Rojo, Héctor Lamadrid-Figueroa, Adriana Mercado-García, Mauricio Hernández-Avila, David Bellinger, Donald Smith, Adrienne S. Ettinger, Joel Schwartz, Lourdes Schnaas

**Affiliations:** Department of Environmental Health Sciences, University of Michigan School of Public Health, Ann Arbor, Michigan, E-mail: howardhu@umich.edu; Centro de Investigación en Salud Poblacional, Instituto Nacional de Salud Pública, Cuernavaca, Morelos, México; Department of Neurology, Children’s Hospital, Harvard Medical School, Boston, Massachusetts; Department of Biology and Environmental Toxicology, University of California, Santa Cruz, California; Department of Environmental Health, Harvard School of Public Health, Boston, Massachusetts; Instituto Nacional de Perinatologia, Mexico City, Mexico

We thank Ronchetti for his comments on our recent article (Hu et al. 2006). We are aware of what he noted was the wide scatter of points surrounding the correlation line of plasma lead in relation to mental development index (MDI) score. Ronchetti suggests that the scatter might improve if one measured the “lake” of maternal lead burden (in this case, maternal bone lead) as opposed to sampling the “river” of maternal lead represented by lead in maternal venous blood, since the “lake” is presumably the ultimate source of maternal circulating lead and therefore fetal lead exposure. We are quite aware of (and have been a proponent of ) this line of reasoning (Hu et al. 1998). Our article (Hu et al. 2006) is just one in a series of reports stemming from a long-running birth cohort study in which we do, in fact, have maternal bone lead measurements (measured noninvasively using K-X-ray fluorescence). In separate analyses we have examined maternal bone lead as an independent predictor of MDI, as well as other outcomes (e.g., Gomaa et al. 2002; Gonzalez-Cossio et al. 1997; Hernandez-Avila et al. 2002; Sanin et al. 2001).

In our study (Hu et al. 2006), our major focus was on comparing the relative contributions of each trimester of lead exposure to fetal neurodevelopment, and we did not include maternal bone lead in our models. If we force maternal tibia lead in the model of first trimester maternal plasma lead and MDI, tibia lead is an independent and significant predictor of lowered MDI and the effect of first trimester plasma lead is attenuated [Table 1; note that the smaller sample size of 113 subjects, compared with 119 subjects in Table 2 of Hu et al. (2006), is due to the smaller number of mothers with bone lead measurements]. This provides some support for maternal bone lead as the best biological marker for predicting lead’s impact on fetal neurodevelopment; however, it does not detract from our observation (Hu et al. 2006) that if one focuses on the individual trimesters in order to examine the question of the greatest window of vulnerability during gestation, the impact of first trimester fetal lead exposure appears to be greater than the impacts of the other trimesters. Moreover, the increase in the variance explained by the model with bone lead compared with the model without bone lead is modest (*R*^2^ values of 0.24 vs. 0.22 when the analysis is restricted to the 113 subjects with bone lead), which translates into a relatively minor improvement in the scattered nature of the points.

In our view, rather than denoting the continued absence of a superior biomarker of lead burden, the scattered nature of the points reflects the general challenge of studying a relationship in which all predictors are measured with a substantial amount of random error; no doubt, there are many other predictors of MDI that remain completely unmeasured (e.g., genetics, nutrition, other potential neurotoxicants). Future studies may be able to improve scatter by improving such measures (and increasing study sample sizes) while, of course, public health measures are hopefully taken to continue reductions in population levels of lead exposure.

## Figures and Tables

**Table 1:** Multivariate model of MDI of offspring (at 24 months of age) using both maternal plasma lead during the first trimester and maternal bone lead (measured in the perinatal period) as markers of prenatal lead exposure (*n* = 113).

Variable	β	*p*-Value
Tibia lead (μg/g)	−0.22	0.03
Maternal plasma lead (μg/L)^a^	−0.97	0.59
Current blood lead (μg/dL)^b^	−0.050	0.22
Sex^c^	2.89	0.18
Mother’s IQ	0.035	0.68
Mother’s age (years)	0.68	< 0.01
Height-for-age *z*-score	2.16	0.12
Current weight (kg)	−2.27	0.02
Intercept	97.96	0.00

Total model *R* = 0.24.

a Measured during the first trimester; log-transformed.

b Measured at 2 years of age.

c Male = 1; female = 2.
